# Natural ventilation reduces high TB transmission risk in traditional homes in rural KwaZulu-Natal, South Africa

**DOI:** 10.1186/1471-2334-13-300

**Published:** 2013-07-01

**Authors:** Melissa Lygizos, Sheela V Shenoi, Ralph P Brooks, Ambika Bhushan, James CM Brust, Daniel Zelterman, Yanhong Deng, Veronika Northrup, Anthony P Moll, Gerald H Friedland

**Affiliations:** 1 University of Colorado School of Medicine, Aurora, CO, USA; 2 Yale University School of Medicine, AIDS Program, New Haven, CT, USA; 3 Harvard University School of Medicine, Boston, MA, USA; 4 Montefiore Medical Center and Albert Einstein College of Medicine, Bronx, NY, USA; 5 Center for Analytical Sciences, Yale University School of Public Health, New Haven, CT, USA; 6 Church of Scotland Hospital, Tugela Ferry, South Africa

**Keywords:** Tuberculosis transmission, MDR/XDR TB, Household, South Africa, Infection control, Ventilation

## Abstract

**Background:**

Transmission of drug susceptible and drug resistant TB occurs in health care facilities, and community and households settings, particularly in highly prevalent TB and HIV areas. There is a paucity of data regarding factors that may affect TB transmission risk in household settings. We evaluated air exchange and the impact of natural ventilation on estimated TB transmission risk in traditional Zulu homes in rural South Africa.

**Methods:**

We utilized a carbon dioxide decay technique to measure ventilation in air changes per hour (ACH). We evaluated predominant home types to determine factors affecting ACH and used the Wells-Riley equation to estimate TB transmission risk.

**Results:**

Two hundred eighteen ventilation measurements were taken in 24 traditional homes. All had low ventilation at baseline when windows were closed (mean ACH = 3, SD = 3.0), with estimated TB transmission risk of 55.4% over a ten hour period of exposure to an infectious TB patient. There was significant improvement with opening windows and door, reaching a mean ACH of 20 (SD = 13.1, p < 0.0001) resulting in significant decrease in estimated TB transmission risk to 9.6% (p < 0.0001). Multivariate analysis identified factors predicting ACH, including ventilation conditions (windows/doors open) and window to volume ratio. Expanding ventilation increased the odds of achieving ≥12 ACH by 60-fold.

**Conclusions:**

There is high estimated risk of TB transmission in traditional homes of infectious TB patients in rural South Africa. Improving natural ventilation may decrease household TB transmission risk and, combined with other strategies, may enhance TB control efforts.

## Background

Airborne infection control is a crucial, often neglected, component of tuberculosis (TB) control [1]. This is particularly true in the era of well-documented primary TB transmission, multidrug-resistant (MDR) and extensively drug-resistant (XDR) TB and in resource-limited settings with high prevalence of TB and HIV [1-6].

Tugela Ferry, in KwaZulu-Natal, South Africa, has been greatly impacted by co-epidemics of HIV and drug-resistant TB [7]. The initial report of the XDR TB epidemic in Tugela Ferry was attributed, in part, to nosocomial transmission [3]. Modeling studies have subsequently suggested that implementation of combined infection control practices could reduce nosocomial transmission [8], but that an increasing proportion of new XDR TB cases would occur in non-healthcare community settings [9]. TB transmission in community settings and among household contacts of drug susceptible, MDR and XDR TB patients has been receiving increased attention [10-17]. The high prevalence of HIV and drug susceptible and resistant TB in the context of low socioeconomic status in rural KwaZulu-Natal, provides a large pool of vulnerable household contacts underscoring the need for infection control practices in this setting [3,7,15-19].

Strategies and guidelines for infection control, including enhanced ventilation, however, have largely concentrated on healthcare settings [1,2,20,21]. Natural ventilation has been shown to be as effective as mechanical ventilation and is particularly attractive in tropical and temperate climates and resource-limited healthcare systems [22]. Previous work in healthcare facilities, but not in household settings, has demonstrated that ventilation, usually measured in air changes per hour (ACH), improves with larger windows and that greater room volume reduces the risk from airborne transmission as estimated using the Wells-Riley model [22-27].

Despite evidence of household and community-based TB transmission – including MDR and XDR TB, little is currently known about air exchange and transmission risk in household or community settings and the potential benefit of ventilation [2]. We evaluated air exchange and the impact of natural ventilation on estimated TB transmission risk in traditional Zulu homes.

## Methods

### Setting

Ventilation was measured in traditional Zulu homes in Tugela Ferry, an impoverished rural area of approximately 180,000 people with high rates of HIV and drug susceptible and resistant TB. Traditional homes, housing multiple family members, are typically one-room round or box-shaped structures, composed of mud or occasionally plaster walls, wooden doors, and topped with a cone-shaped thatch roof or slanted sheet of metal, respectively. Windows, if present, are usually small compared to the size of the home.

### Home measurements

Traditional homes of community members were volunteered and no incentive was given. We recorded the following structural characteristics: building shape and materials; roof materials and thickness; wall thickness and height; wall and ceiling height; room width and volume; door area; windows number, area and position; cross-ventilation; presence, size and type of other ventilation spaces. We defined cross-ventilation as pairs of opposing windows or windows across from the door. In round homes, windows and doors were considered opposing if at an angle of greater than 135 degrees relative to each other.

To account for substantial variability in both the room size and window area amongst homes, a Window-to-Volume Ratio was created, representing the space available for ventilation per volume of air that would need to travel through that space to create an air exchange. This ratio denotes the geometric differences in the conical thatched and flat metal roofed traditional homes.

### Environmental measurements

The following environmental variables were recorded at the initiation of experimentation and hourly throughout the testing session: outside and inside temperature; wind speed at the door, window, and 10 meters from the home where wind flow was unobstructed; relative humidity, and direction of air flow at the door. An AZ-8912 anemometer (Laesent International Co., China) was used to measure all variables with the exception of direction of air flow, which was visualized using the smoke from burning incense sticks.

### Ventilation measurements

A carbon dioxide (CO_2_) concentration-decay technique was used to measure ACH during late summer through winter [26]. All windows and doors were closed or blocked off, and a baseline CO_2_ level in parts per million (ppm) was measured. CO_2_ was released from a pressurized cylinder tank and the concentration was raised to >8,000 ppm. A fan mixed the air for about one minute to provide an even concentration of CO_2_ throughout the room. The ambient CO_2_ concentration was measured every 30 seconds using a centrally placed analyzer (Bacharach 2815, USA). After the CO_2_ concentration decay rate with closed door and windows was established, or after approximately 5 minutes, the testing conditions were changed (e.g. windows partially opened); 30–60 seconds were allowed for the changes in ventilation to occur and for a steady-state to be reached, at which time CO_2_ measurements were retaken; CO_2_ decay was captured under the following conditions: windows and door closed; windows partially open (door closed); windows fully open (door closed); window and door both open. In each condition CO_2_ concentration was recorded until it fell to within 200 ppm of the baseline level, concluding that trial. ACH was calculated as the gradient of the best-fit line through a plot of the natural logarithm of CO_2_ concentration in ppm plotted against time in hours.

### TB risk estimation

To estimate the risk of TB infection we used the Wells-Riley equation [ *C* = *S*(1 − *e*^− *Iqpt*/*Q*^) , see legend Table 1] [23,24]. When possible, we used previously established values for variables to facilitate comparison between this and other studies [22]. We designated time of exposure (*t*) as 10 hours, based on the amount of time a person might spend inside a home overnight in close contact with an infectious TB patient.

**Table 1:** ACH and estimated TB risk in predominant home types

	**All homes**	**Box-shaped, Metal-roofed**	**Round-shaped, Thatch-roofed**	
**ACH**	**Mean (SD)**	**Mean (SD)**	**Mean (SD)**	**p-value**
Closed	3 (2.9)	3 (2.7)	3 (3.3)	0.50
Windows Open	9 (7.1)	13 (8.1)	5 (2.9)	0.01
Windows & Door	20 (13.1)	27 (9.7)	13 (12.8)	0.01
Open				
*TB Risk (10h exposure)				
Closed	55.4 (27.8)	58.3 (24.7)	52.5 (31.5)	0.62
Windows Open	21.5 (14.1)	24.7 (18.1)	18 (8.4)	0.32
Windows & Door	9.6 (4.7)	8.9 (3.6)	10 (5.6)	0.44
Open				

*SD* standard deviation.

Wells Riley equation: *C* = *S*(1 − *e*^− *Iqpt*/*Q*^) where C = number of new cases, S = number of susceptible individuals, I = number of infectors (presumed to be 1 per household), q = number of infectious quanta produced per hour per infector (assumed to be 13 based on previous studies) [22], p = pulmonary ventilation rate of susceptible individuals (0.6 m3/h, previously established), t = exposure time of susceptible individuals, Q = absolute room ventilation (ACH*room volume). The probability of a new case was C/S.

### Statistical methods

Descriptive statistics summarized the data; box-plots of ACH were created for each ventilation condition. ‘Windows partially open’ and ‘windows fully open’ were collapsed as there was no statistical difference between these conditions. Evaluations of ACH and percent TB risk, were performed using mixed effects regression modeling, where each home was treated as a random effect and the repeated nature of the observations within a home was taken into account. ACH during the closed condition was a covariate. Each predictor of interest was considered in the models independently and significant variables at p < 0.05 were then considered in a multivariate model. A variance inflation factor (VIF) was used to assess multicolinearity between variables in the multivariate models: *VIF* = 1/[1 − *r*^2^] , where *r* is bivariate Pearson correlation coefficient; if variables were found to be highly correlated they were not considered together in the multivariate models. Generalized estimating equations (GEE) were utilized to evaluate significant predictors of the probability of achieving ACH ≥12. Significance was established with alpha = 0.05 and adjusted for multiple comparisons using the Bonferroni approach. Data were analyzed using SAS 9.2 (Cary, NC).

### Ethics statement

Ethical approval was obtained from University of KwaZulu-Natal, Durban, South Africa, Albert Einstein College of Medicine, Bronx, New York, USA, and Yale University School of Medicine, New Haven, CT, USA. Verbal consent was obtained from household members. The data collected focused exclusively on physical structures and did not pertain to any individual’s personal or health information.

## Results

### Home, environmental, and ventilation measurements

**Figure 1:**
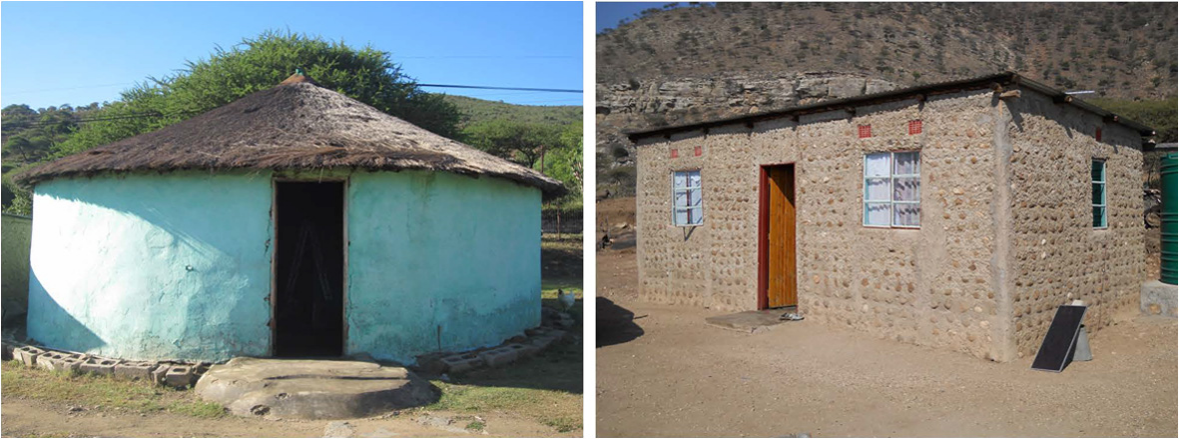
**Two predominant types of traditional home construction in Tugela Ferry.** A. Round-shaped home with thatched roof B. Box-shaped home with metal roof.

Two hundred eighteen ventilation measurements were conducted in 24 homes; 12 round-shaped huts with thatched roofs and 12 box-shaped homes with metal roofs (Figure 1). The structural and environmental characteristics of the homes are shown in Table 2. Compared to box-shaped, round homes had greater room volumes (91.3 m^3^ vs. 36.6 m^3^, p < 0.001), were more likely to have cross-ventilation (10 vs. 2 homes, p < 0.01), but had a smaller Window-to-Room Ratio (8*10^-3^ m^-1^ vs. 27*10^-3^ m^-1^, p < 0.01). The ambient indoor and outdoor temperatures and the wind speed are shown in Table 2. Air was flowing in through the door in 14 homes (58%), out the door in 7 (29%), and had no clear direction in 3 homes (13%).

**Table 2:** Home Structural Characteristics (n = 24)

	
Shape	
Round	12 (50%)
Box	12 (50%)
Roof Materials	
Thatch	12 (50%)
Metal	12 (50%)
Number of windows	
0	4 (17%)
1	6 (25%)
2	10 (42%)
3	2 (8%)
4	2 (8%)
Cross Ventilation	
None	12 (50%)
Window-Window	9 (38%)
Window-Door	6 (25%)
Window Area, mean (SD)	
Single window [m^2^]	0.5 (0.2)
Total window area for home [m^2^]	0.9 (0.7)
Door Area [m^2^], mean (SD)	1.5 (0.3)
Floor Area [m^2^], mean (SD)	22.7 (12.4)
Room Volume [m^3^], mean (SD)	63.9 (38.5)
Wind Speed, baseline [m/s], mean (SD)	
At door	0.4 (0.4)
At window	0.7 (0.5)
Unobstructed	1.4 (0.6)
Temperature, baseline [°C], mean (SD)	
Inside	25.2 (3.6)
Outside	28.2 (5.0)
Temperature difference (mean)	3.4 (3.0)

SD = standard deviation.

**Figure 2:**
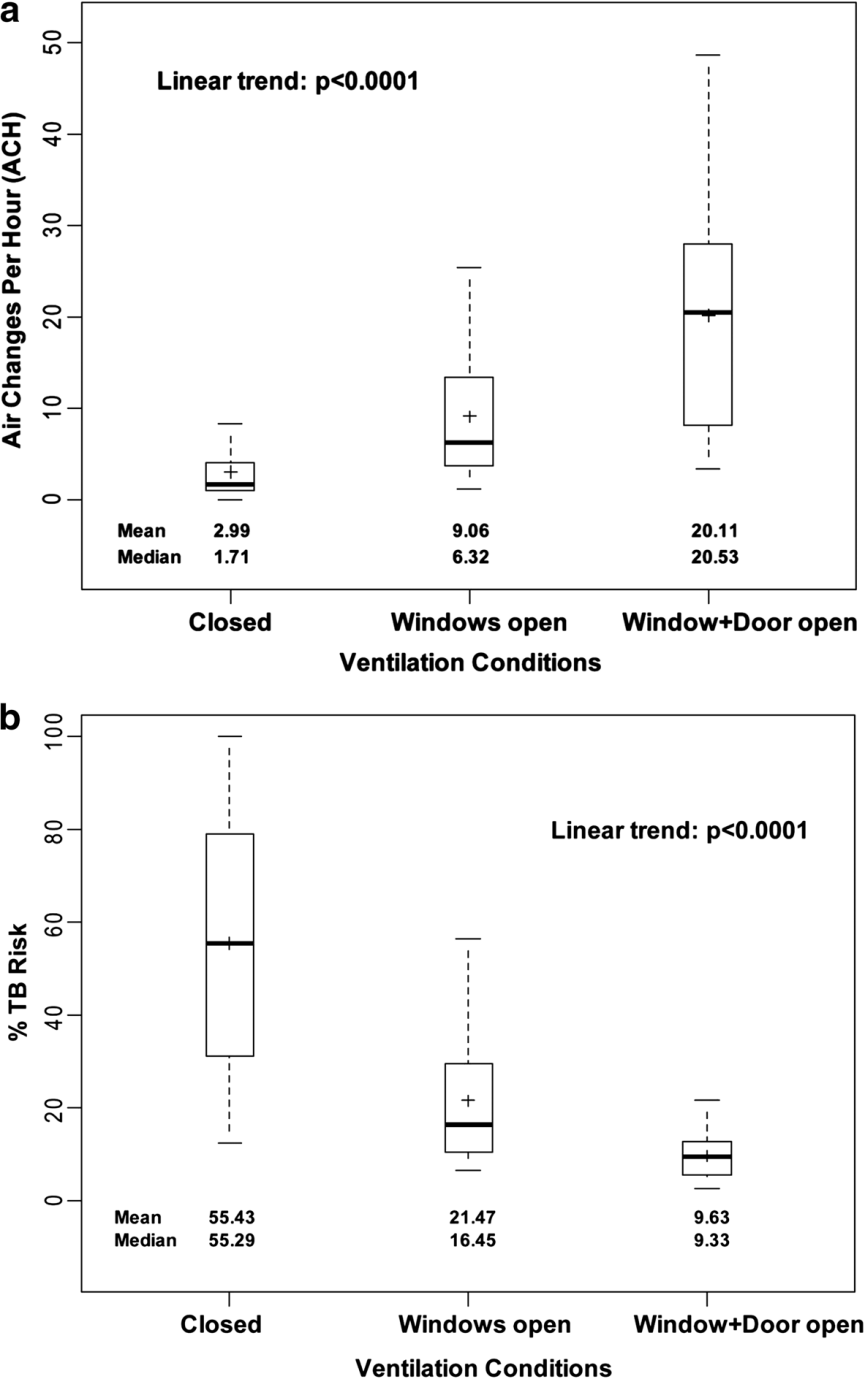
**ACH and Estimated TB Risk under different ventilation conditions.** Box-plot of ACH and estimated TB risk from the various ventilation conditions tested: (1) baseline with windows and door closed, (2) windows open, and (3) windows and door open. Mean ACH and TB risk for each condition is marked with “+”. Median ACH and TB risk is marked by the solid horizontal line, with the upper and lower ends of the box representing the limits of the interquartile range (IQR). The dotted lines represent the range of values. a. ACH b. Estimated TB Risk.

Among all homes, the mean ACH improved when windows were opened and improved further when windows and door were both opened (p < 0.0001) (Table 1, Figure 2a). Of note, there was no significant difference in ACH under closed conditions when any extra ventilating spaces, such as vents, remained open or were covered. Although there was no difference in baseline ACH, ventilation was more favorable in box-shaped metal roof homes than in round thatched roof homes as windows and door were opened (p = 0.01) (Table 1).

### TB risk estimation

**Figure 3:**
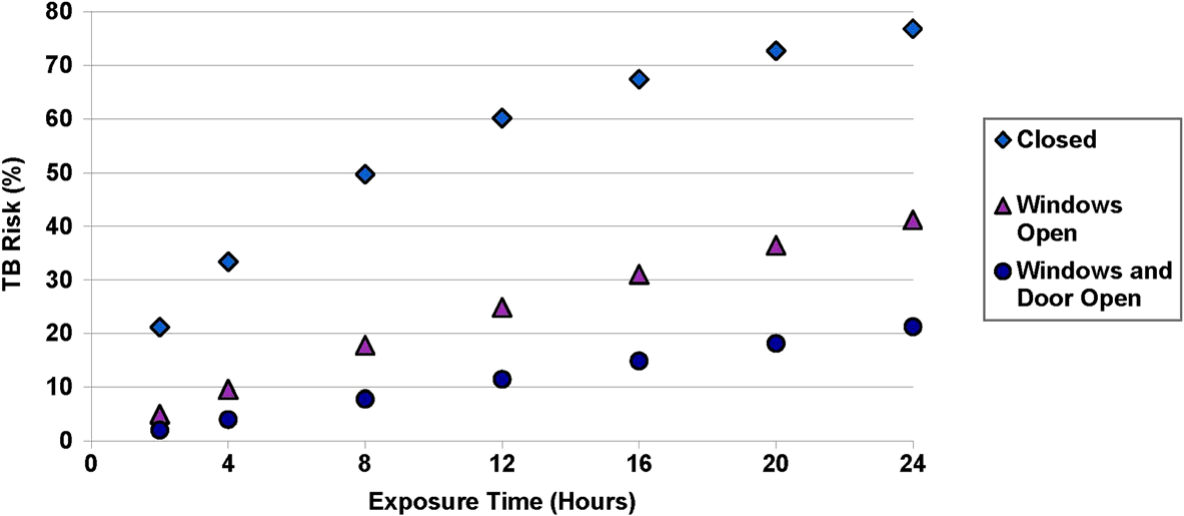
**Estimated Risk of TB Infection by Exposure Time.** Risk of TB infection after various durations of exposure to an active TB case under different ventilation conditions, as estimated using the Wells-Riley equation. Diamond: windows and door closed; Triangle: windows open; Circle: windows and door open together.

The estimated risk of TB transmission after ten hours of exposure to an infectious TB patient with windows and door closed was 55.4% (SD = 27.8%). This risk dropped significantly upon opening windows (21.5%, SD = 14.1%, p < 0.001), and further upon opening windows and door together (9.6%, SD = 4.7, p < 0.001) (Table 1, Figure 2b). The estimated risk of TB infection increased in parallel to exposure time (p < 0.001) (Figure 3). Despite the differences in ACH, there was no significant difference in estimated TB transmission risk under any condition between the two main home types (Table 1). Notably, the estimated risk with 2 hours of exposure in a closed room approximates that at 24 hours with windows and doors open (Figure 3).

### Predictors of ACH

Univariate analyses identified factors that were significantly associated with ACH (Table 3). Multivariate analysis demonstrated a model (Table 4) in which ACH was dependent on the ventilation conditions, as ACH increased from windows closed to windows open to windows and door open, as well as on the Window-to-Volume Ratio (ACH increased by 0.4 for every unit increase in the ratio, F-value = 24.2, p < 0.0001) (Table 4). The enhanced ventilation increased the odds of achieving a minimum of 12 ACH by 60-fold (Table 4).

**Table 3:** Univariate analyses identifying factors associated with ACH in traditional Zulu homes

**Variables**	**Coefficient**	**p value(N = 24)**
Height of walls	11.57	0.01
Width of room	−4.57	0.02
Floor area	−0.38	0.01
Volume of room	−0.12	0.02
Surface area of walls	−0.13	0.62
Door area	8.03	0.21
Window number	2.79	0.09
Window area	5.66	0.05
Combine area of wind/door	5.44	0.02
Wind speed at door	4.08	0.45
Wind speed 10m	0.82	0.83
House Shape	9.69	0.005
Materials of roof	10.43	0.005
Metal vs. Thatch		
Other ventilation space	−1.80	0.75
Absent vs. Present		
Window area/wall surface	372.65	0.0024
Window area/floor area	145.68	0.0001
Window/volume	414.23	<0.0001
Temperature		
Inside	0.03	0.96
Outside	−0.43	0.31
Difference	−1.00	0.10
Relative humidity	0.12	0.49
Smoke direction		
In	5.46	0.40
Out	8.15	0.26

**Table 4:** Adjusted mixed model analysis associated with improved ACH in traditional Zulu homes

	**Model 1**			
**Variable**	**Parameter Estimate (SE)**	**p-value**^**1**^	**Odds Ratio (95% CI)**	**p-value**^**2**^
ACH in closed condition	1.7 (0.5)	<0.01	1.8 (1.2,2.7)	<0.01
Ventilation Condition				
Window Open	10.9 (2.1)	<0.0001	66.6 (5.0,790.8)	<0.01
Window & Door Open				
Window-Volume Ratio	0.4 (0.02)	<0.0001	1.5 (1.1,2.0)	<0.01

^1^ Obtained from mixed effects regression.

^2^ Obtained from GEE.

The finding that window-volume ratio is a significant factor may be less helpful for the average community member in our impoverished resource limited setting to implement. A second equally strong model (Table 5) similarly demonstrated that ACH is dependent on ventilation conditions (windows closed, windows open, windows and door open), but instead of the window-volume ratio, this model demonstrates that the key factors associated with ACH are home shape (box-shape vs. round-shape, F-value = 20.3, p = 0.0002) and the number of windows (F-value = 11.1, p < 0.01).

**Table 5:** An alternative adjusted mixed model analysis associated with improved ACH

	**Model 2**	
**Variable**	**Parameter Estimate (SE)**	**p-value**^**1**^
ACH in closed condition	1.9 (0.5)	<0.01
Ventilation Condition		
Window Open	11.5 (2.1)	<0.0001
Window & Door Open		
Type of Home		
Simple Box	12.2 (2.7)	<0.001
Simple Round		
Number of windows	4.8 (1.4)	<0.01

^1^ Obtained from mixed effects regression.

## Discussion and conclusions

We measured and evaluated air exchange, estimated TB transmission risk, and the impact of natural ventilation in traditional homes and huts in rural South Africa. We believe that this is the first study to perform such measurements and to quantitate TB transmission risk at the household level in a high prevalence HIV and TB and drug resistant TB rural setting. We found low baseline ventilation and an extremely high estimated risk of TB transmission (9.6-55.4%) in household settings, where index patients with TB interact frequently with vulnerable family members and other community members, including those possibly HIV infected in this high prevalence HIV area. We also found and quantitated simple measures in the household setting that could significantly improve ventilation and reduce transmission risk in the household setting.

Studies of TB transmission have focused on health care settings, and evidence based airborne infection control strategies have been developed to reduce transmission in hospitals and clinics. Although there is increasing recognition of TB transmission occurring in community settings, particularly households, the scientific and public health communities lack evidence to inform infection control strategies in this setting [2,10-17]. The WHO recommendation of ACH ≥ 12 is based on estimates in health care settings [1]; there are no clear data to inform recommendations for non health care settings in endemic TB regions [2]. The present study provides important new descriptive information to help understand air exchange and TB transmission risk in the household setting in rural sub-Saharan Africa and also demonstrates that the simple procedure of opening existing windows and doors can significantly reduce TB transmission risk. Though these findings may be intuitive, the provision of quantitative estimates of ACH and TB transmission risk can be used to inform patients, communities, programs and future household construction and help spur development and implementation of airborne infection control strategies suitable for community settings. Ventilation strategies may be a useful adjunctive tool to case finding, rapid diagnostics, improving treatment outcomes, and ART expansion.

Previous studies have shown that the area contributing to ventilation, such as the area of an open window, have an impact on ventilation; even intermittent window opening may significantly improve ventilation and air quality within a room [28]. However, our evaluation also demonstrated the importance of the room volume. Though box-shaped metal roof homes had higher ACH, this was offset by the larger volume of round thatch-roof homes with resultant similar estimated TB transmission risk. Multivariate analyses demonstrated that enhancing the ventilation conditions by opening windows and doors and a greater window/volume ratio were associated with better ACH. In a less technically complex, equally strong model that could be more easily utilized to educate community members, opening doors and windows, box-shaped metal roof homes (compared to round thatched roof), and increased number of windows were associated with improved ACH. Both models suggest that greater opportunities for air flow and increased area available for ventilation are important determinants of ACH in this setting. Community members may be able to plan renovations or new homes with box-shaped, metal roofs and an increased number of windows.

While previous studies of natural ventilation in healthcare settings have emphasized the direction of air movement, this does not likely have as much consequence in our setting as most homes do not have adjoining rooms into which infectious particles may enter after leaving the room in question. Temperature, wind speed and direction, and humidity were not significant predictors, possibly due to differences in climate.

In our study, the ventilation improved incrementally as the area contributing to ventilation increased, though many homes had only a few small windows, limiting substantial increases. In most homes, only when windows were opened in combination with the door was the WHO recommended 12 ACH threshold reached [1,21]. The full success of this intervention may require increasing the number and/or size of windows and ensuring that windows and doors remain open. Both represent challenges in rural Africa where resources for construction are limited and closing windows and doors at night for safety and/or to reduce insect exposure is common. Information regarding household usage patterns, acceptability of infection control interventions, and barriers to implementation are needed to inform the development and evaluation of more comprehensive behavioral and community-oriented airborne infection control strategies.

We recognize several limitations in this study. First, the two home construction types examined are typical of rural KwaZulu-Natal, South Africa. Thus, the transmission risk data may not be fully applicable in other resource-limited settings, even in sub- Saharan Africa. For example, in another study in an urban township in South Africa use of public transportation appeared to confer great TB transmission risk than in the household setting [12]. We do believe that the data gathered in our study are sufficiently similar to those in other rural settings in sub-Saharan Africa to have broader relevance. Secondly, natural ventilation is feasible in tropical climates or in temperate climates only during warmer seasons and limited in colder climates. Additionally, though being the best currently available model for TB transmission and one that has been used in similar studies, the Wells-Riley model provides a theoretical, not a true, assessment of risk. The equation utilizes many important factors, but some such as the number of infectious particles produced by a patient; or proximity of the infectious and susceptible individuals to each other, are not taken into account. Furthermore, the equation measures steady exposure over a finite time period, 10 hours in this study, whereas TB transmission risk, in reality, encompasses multiple exposures and may be cumulative, particularly in household settings.

Further work is now needed to apply the information gained from this study on a broader public health level, to develop strategies to reduce TB transmission in this and other rural communities heavily impacted by drug susceptible and drug resistant TB. Although there are economic and logistical barriers, the cost and challenges of airborne infection control neglect should be calculated in the context of missed opportunities for potential prevention of morbidity and mortality and secondary TB transmission.

## Competing interests

The authors declare that they have no competing interests.

## Authors’ contributions

ML, SVS, RPB, JCMB, APM, GHF designed the study. ML, RPB, AB performed the experiments. ML, SVS, RPB, AB, DZ, VN, YD, GHF analyzed the data. ML, SVS, RPB, JCMB, VN, APM, GHF wrote the manuscript. All authors read and approved the final manuscript.

## Pre-publication history

The pre-publication history for this paper can be accessed here:

http://www.biomedcentral.com/1471-2334/13/300/prepub
